# Comparative Evaluation of Three Access Cavity Preparation Techniques on Root Canal Instrumentation Using Micro-CT: An In Vitro Study

**DOI:** 10.7759/cureus.66424

**Published:** 2024-08-08

**Authors:** Mrunmayee V Khare, Ranjith Kumar Sivarajan, Vijay Venkatesh

**Affiliations:** 1 Department of Conservative Dentistry and Endodontics, Sri Ramaswamy Memorial (SRM) Kattankulathur Dental College and Hospital, SRM Institute of Science and Technology (SRMIST), Chennai, IND

**Keywords:** debris formation, untouched surfaces, apical transportation, trunatomy files, 3d guided access cavity, contracted endodontic access, traditional endodontic access

## Abstract

Background

Access cavity preparation is a crucial step in nonsurgical root canal treatment. Recent advancements in access cavity designs focus on preserving maximum tooth structure while ensuring sufficient access to canal orifices for effective cleaning and shaping, resulting in minimally invasive procedures. However, there is limited information on the impact of three-dimensional (3D)-guided access cavity preparation in molars. A literature review found no prior studies comparing the effects of various access cavity preparation techniques on apical transportation, untouched surfaces, and debris formation within the canal.

Objective

The objective of this study is to compare and evaluate the effects of three different access cavity techniques on apical transportation, untouched surfaces, and debris formation within the root canal.

Material and methods

Thirty extracted permanent mandibular first molars were selected and randomly assigned to three groups for this study: Group I received 3D-printed static guided cavity preparation, Group II underwent conservative access cavity preparation, and Group III was subjected to traditional access cavity (TAC) preparation. The mesial canals in all samples were cleaned and shaped using TruNatomy files. Preoperative and postoperative micro-CT imaging was performed on each sample to assess the effects of the different access cavity preparation techniques on apical transportation, untouched surfaces, and debris formation within the root canal.

Results

The study found that Group I, which used 3D-printed static guided cavity preparation, exhibited significantly less apical transportation compared to Groups II and III, with mean differences of -0.1677 and -0.2079, respectively. Debris accumulation was similar across all groups, with mean values of 0.928 ± 0.824 for Group I, 0.751 ± 0.495 for Group II, and 0.938 ± 0.681 for Group III, indicating no significant impact of cavity preparation type on debris levels. For untouched canal surfaces, Group III (TAC preparation) had the fewest untouched surfaces, with mean differences of 3.0380 and 3.9020 compared to Groups II and I, respectively.

Conclusions

While TAC preparation reduces substantial tooth structure, it shows higher instrumentation efficacy and better cleaning of the root canal system. However, in complex cases where tooth structure preservation is crucial, guided access cavity preparation provides an effective balance between structural conservation and adequate canal access. This approach offers a tailored solution, optimizing treatment outcomes based on the specific clinical scenario.

## Introduction

Endodontic access cavity preparation is a critical aspect of successful nonsurgical root canal treatment [1]. Traditional access cavity (TAC) preparation, based on designs proposed by Black in 1908 and adapted by Ingle in 1965, aims to provide direct, straight-line access to the pulp chamber by complete de-roofing of the pulp chamber and any dentinal protrusions [2]. This method achieves unhindered access to the root canal orifices and the apical foramen, but the extensive removal of tooth structure significantly reduces the tooth’s fracture resistance, increasing its susceptibility to fracture under functional loading due to enhanced cuspal flexure [3-5].

To address these drawbacks, the concept of minimally invasive access cavity preparation was introduced. This conservative approach focuses on preserving as much tooth structure as possible, particularly the pericervical dentin, which has been shown to improve fracture resistance [6-9]. Conservative or contracted endodontic access cavities have demonstrated better outcomes in terms of maintaining tooth integrity while still allowing sufficient access for cleaning and shaping the root canal system [5,8].

Pulpal calcifications, whether partial or complete, are common in permanent teeth and pose significant challenges during root canal treatment. These calcifications can lead to complications such as perforations or deviations, adversely affecting the prognosis of the treatment [10]. “Guided endodontics” is an innovative technique designed to overcome these challenges by providing precise guidance for access cavity preparation, making it particularly useful in difficult cases [11]. Recent studies have shown that guided access preparations are more predictable and faster for locating and negotiating canals, with a significant reduction in substance loss compared to traditional methods [11,12].

Research by Blauhut and Sonntag indicated that conservative access cavity (CAC) designs might lead to inaccurate interpretations of the number and form of canal orifices when compared to TAC preparations [13]. However, some studies have found no significant differences in instrument efficacy or biomechanical preparation between conservative and traditional techniques [5,14]. Connert et al. compared guided endodontics with conventional access cavity preparation and found that guided endodontics resulted in significantly less substance loss [11]. Studies by Rover et al. and Krishan et al. revealed that distal canals of mandibular first molars showed a higher percentage of untouched walls post-instrumentation with contracted access cavities than with conventional access cavities [7,15,16]. Furthermore, Eaton et al. and Zhang et al. reported that the maximum curvature angle of the canal was higher in teeth with conservative access cavities compared to traditional access cavities, leading to an increased risk of canal transportation during instrumentation [17,18].

The use of micro-CT imaging has become an effective technique for assessing the quality of root canal shaping. Micro-CT provides high-resolution images that enhance the accuracy of qualitative and quantitative measures of the root canal system, allowing for a detailed analysis of shaping parameters. Micro-CT scanning uses a higher radiation dose and a longer scanning time and is hence often used in laboratory research [19].

Despite the advancements and studies conducted, there remains a lack of comprehensive research comparing the effects of three different access cavity preparation techniques-traditional, conservative, and guided-on apical transportation, untouched surfaces, and debris formation in the canal [7,8]. Therefore, the present study aims to fill this gap by evaluating and comparing these three access cavity techniques, focusing on their impact on apical transportation, untouched canal surfaces, and debris formation within the root canal system.

## Materials and methods

The study was conducted at Sri Ramaswamy Memorial (SRM) Kattankulathur Dental College and Hospital, SRM Institute of Science and Technology (SRMIST), Chennai, India. Following ethical committee approval no. 2316/IEC/2020 from the SRMIST, sample size determination was performed using the following formula:

$n= \frac{Z\alpha ^{2}\cdot sd^{2}}{d^{2}}$,

where n = number of samples, 𝑧∝ = type 1 error, sd = standard deviation, and d = precision.

Thirty extracted human permanent mandibular first molars were selected. The inclusion criteria included teeth without caries or defects on the occlusal surface and canal curvatures between 10 and 20 degrees. Teeth with extensive restorations or incomplete roots were excluded. The selected samples were randomly divided into three groups (10 samples each), disinfected with sodium hypochlorite, and preserved in a 0.5% chloramine T solution for one week. Micro-CT scanning was performed on all samples using a SKYSCAN 2214 with Software Version 1.8. Scanning was conducted at 80 kV and 90 μA with a 360° rotation in 0.2° steps and a 1 mm-thick aluminum filter to reduce beam hardening.

Each group employed a different access cavity preparation technique: Group I used a three-dimensional (3D) printed static guide for the access opening, Group II implemented a CAC preparation, and Group III followed a TAC preparation method.

Preoperative micro-CT analysis

Analysis of Debris Accumulation

To calculate debris accumulation, the volume of the mesiobuccal canal space was first measured using preoperative micro-CT scanning. This baseline measurement allowed for the assessment of debris accumulation following the root canal procedures.

Analysis of Apical Transportation

**Figure 1:**
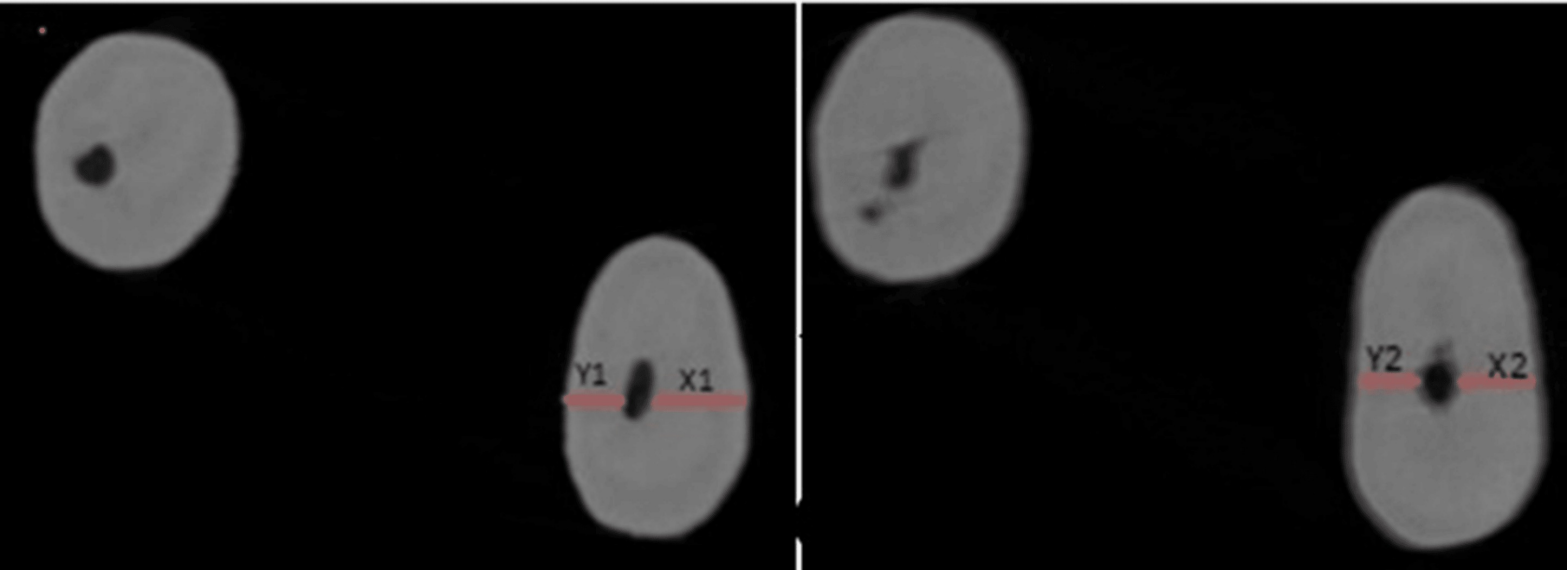
Analysis of apical transportation X1: shortest distance from the end of the mesiobuccal canal to the mesial surface of the mesial root pre-instrumentation; Y1: shortest distance from the end of the mesiobuccal canal to the distal surface of the mesial root pre-instrumentation; X2: shortest distance from the end of the mesiobuccal canal to the mesial surface of the mesial root post-instrumentation; Y2: shortest distance from the end of the mesiobuccal canal to the distal surface of the mesial root post-instrumentation

To analyze apical transportation, measurements were taken using the formula described by Kırıcı et al.:

(X1−X2)−(Y1−Y2),

where X1 is the shortest distance from the end of the mesiobuccal canal to the mesial surface of the mesial root pre-instrumentation, Y1 is the shortest distance from the end of the mesiobuccal canal to the distal surface of the mesial root pre-instrumentation, X2 is the shortest distance from the end of the mesiobuccal canal to the mesial surface of the mesial root post-instrumentation, and Y2 is the shortest distance from the end of the mesiobuccal canal to the distal surface of the mesial root post-instrumentation, as shown in Figure 1 [20].

Analysis of Untouched Canal Surfaces

The untouched canal surfaces were quantified as a percentage by calculating the static voxels post-instrumentation divided by the total number of voxels present on the surface of the mesiobuccal canal [21].

Access cavity preparation

Group I: Access Opening With a 3D-Printed Static Guide

**Figure 2:**
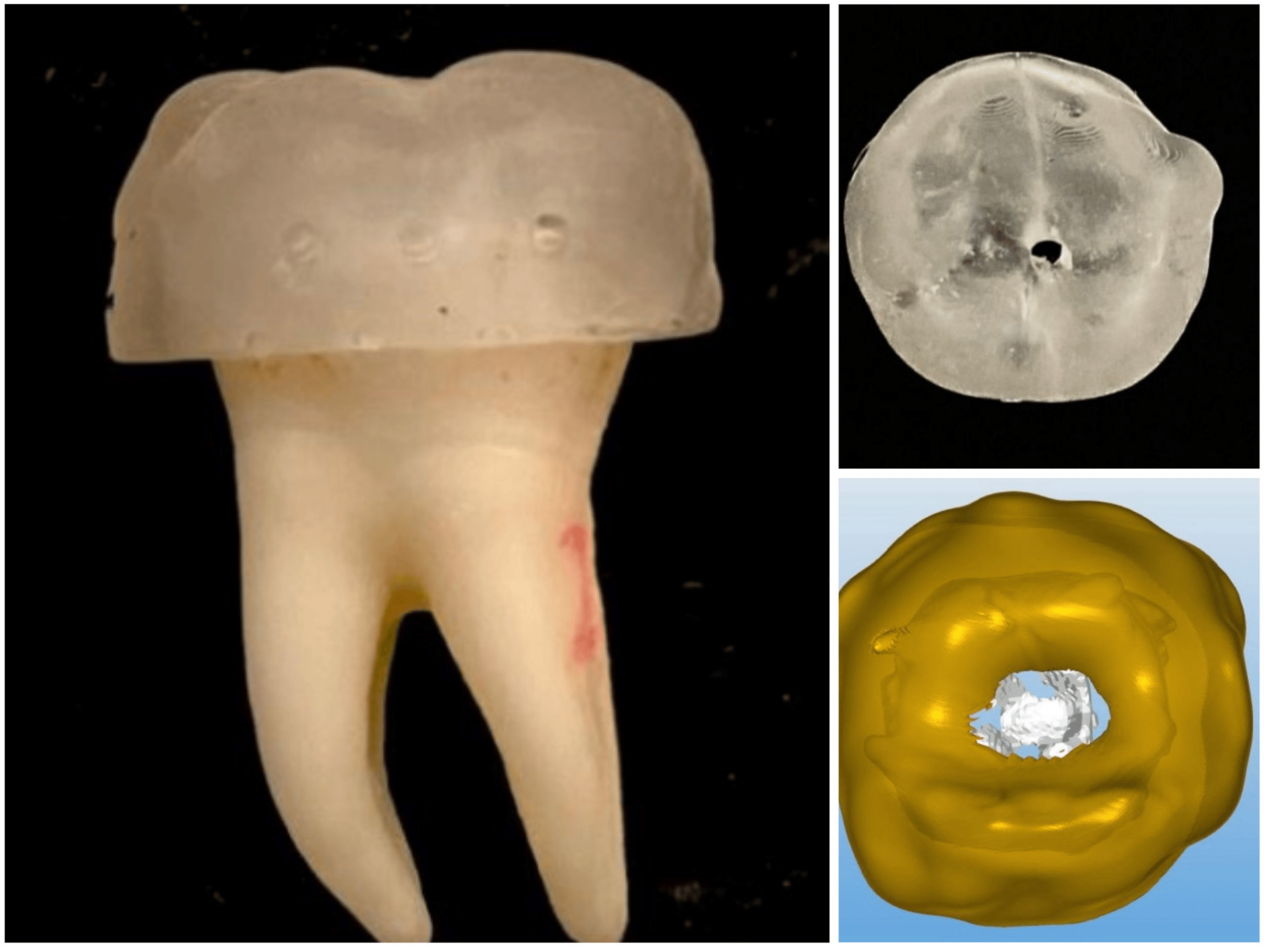
Three-dimensional printed static guide

The 3D-printed static guide for this group was designed using Materialise software and fabricated using a transparent resin material on a Formlabs 2 SLA printer, as shown in Figure 2.

**Figure 3:**
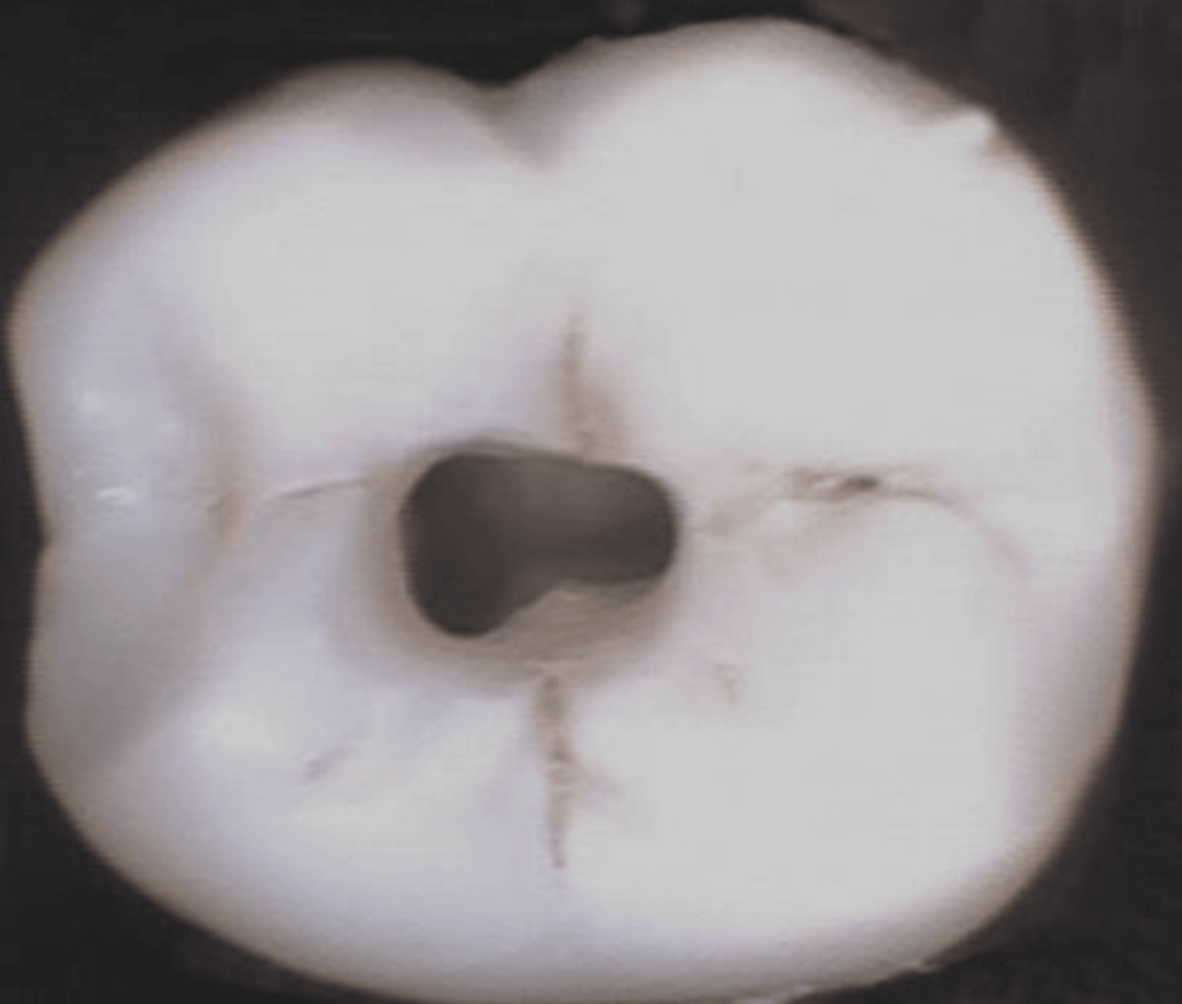
Access cavity preparation using a 3D-printed guide 3D, three-dimensional

The access cavity preparation was performed with a cylindrical diamond bur with rounded edges (837 KR; Intensiv SA, Montagnola, Switzerland) and a Muller bur (Gebr Brasseler GmbH & Co. KG). The entire procedure was conducted under a dental operating microscope at 1× magnification by a single operator to ensure precision. This method aimed to provide a highly accurate and reproducible approach for the preparation of the access cavity, enhancing the quality and consistency of the treatment outcomes, as shown in Figure 3.

Group II: CAC Preparation

**Figure 4:**
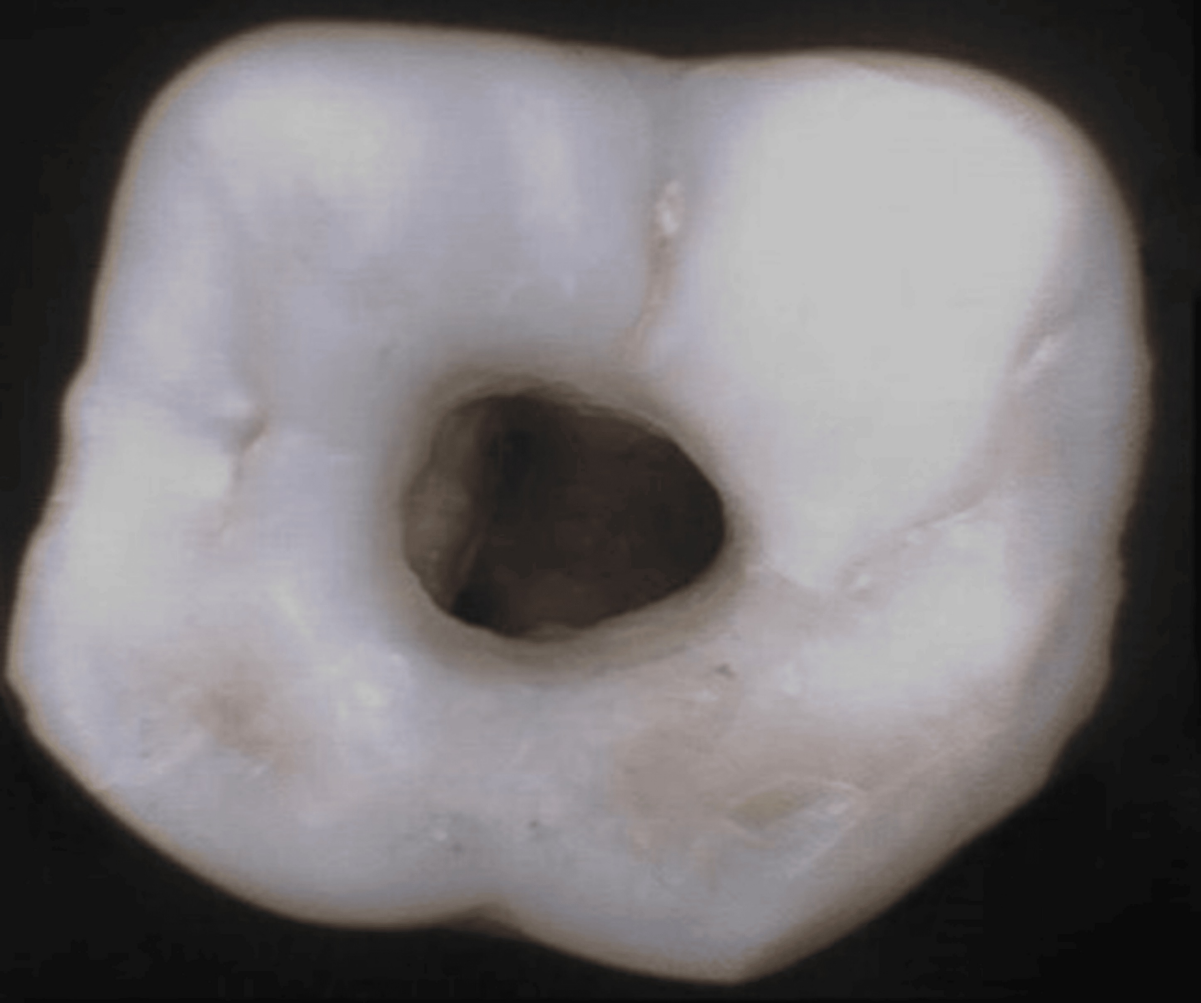
CAC preparation CAC, conservative access cavity

In this group, access cavities were extended apically while maintaining the roof of the chamber to preserve pericervical dentin. The conservative endodontic access cavity preparation was performed using Endo Access Bur (Dentsply Maillefer) and Endo Z burs (Dentsply Sirona, Ballaigues, Switzerland). The procedure was carried out under a dental operating microscope at 1× magnification by a single operator to ensure careful preservation of tooth structure while allowing adequate access for cleaning and shaping the root canal system. This technique focuses on conserving tooth structure, thereby potentially enhancing the tooth’s fracture resistance and overall integrity, as shown in Figure 4.

Group III: TAC Preparation

**Figure 5:**
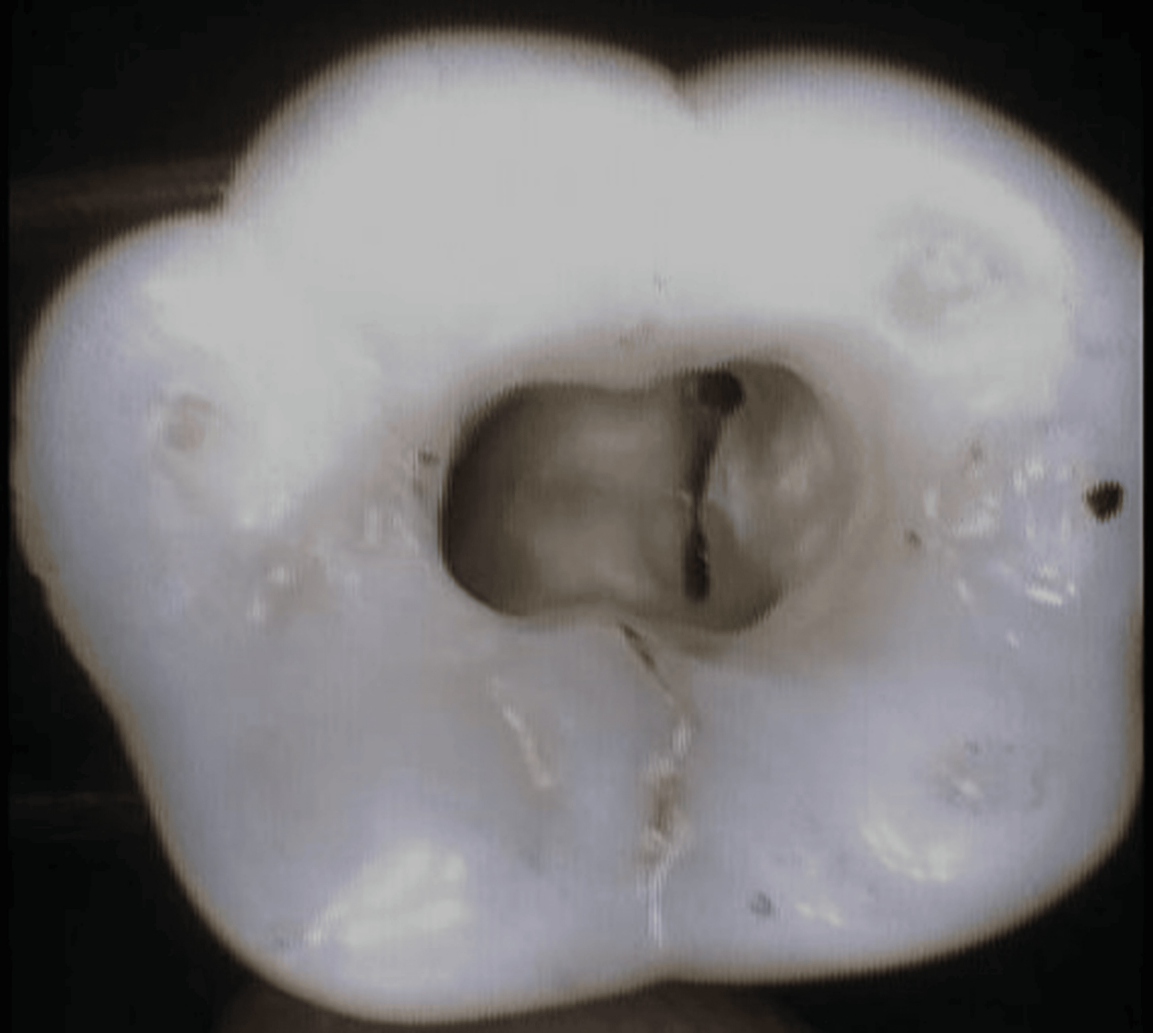
TAC preparation TAC, traditional access cavity

A straight-line access was achieved in this group to ensure that all canals could be visualized within the same field of view. The traditional endodontic access cavity preparation was performed using Endo Access Bur (Dentsply Maillefer) and Endo Z burs (Dentsply Sirona). This procedure was also conducted under a dental operating microscope at 1× magnification by a single operator to facilitate thorough visualization and instrumentation of the canals, as shown in Figure 5.

Biomechanical preparation

Following the access cavity preparation, a uniform cleaning and shaping protocol was implemented. The orifices of the canals were located using a DG-16 probe. To begin, the patency of the mesiobuccal canal was determined using a #10 K file. The working length was then established using a #15 K file, which was measured by subtracting 1 mm from the length at which the file was visible at the root apex.

Instrumentation of the canals was carried out using TruNatomy files (Dentsply) following the manufacturer’s guidelines. The Master Apical File size used was 26/04. During the instrumentation process, the canals were irrigated with 5.25% sodium hypochlorite (NaOCl) after each file. This was accompanied by intermittent irrigation with saline to effectively flush out the debris from the canals.

Postoperative micro-CT analysis

Analysis of Apical Transportation

Postoperative micro-CT analysis assesses surgical accuracy by comparing preoperative and postoperative scans to detect deviations in the root apex, known as apical transportation. The analysis was done using Dataviewer. High-resolution preoperative scans establish a baseline, which is compared to postoperative images using specialized software.

Analysis of Debris Accumulation

In this study, the volume of debris accumulation in each specimen was analyzed by calculating it as a percentage of the total volume of the mesiobuccal canal system. Analysis was done using CTan. To do this, the debris was considered a material with a density comparable to dentine, present within the instrumented canal sections that had initially been filled with air. This approach allowed for a clear and consistent comparison of debris volume relative to the total canal volume.

Analysis of Untouched Canal Surfaces

The percentage of untouched canal surfaces was determined by measuring the static voxels, which represent the areas of the canal surface that remained untouched during the procedure. Analysis was done using CTan. This was achieved by dividing the number of static voxels by the total number of voxels present on the canal surface.

## Results

Data regarding apical transportation (mm), debris accumulation (mm^3^), and untouched canal surfaces (%) in experimental groups was entered into Microsoft Excel (Microsoft Corporation, Redmond, WA, USA) and analyzed using IBM SPSS Statistics for Windows, Version 20.0 (Released 2011; IBM Corp., Armonk, NY, USA). The data was investigated for normality using the Shapiro-Wilk test, and it showed a normal distribution. Descriptive statistics were derived as the mean and standard deviation. The apical transportation, debris accumulation, and untouched canal surfaces between the groups were analyzed using a one-way ANOVA followed by multiple comparisons with Tukey’s honestly significant difference test (α = 0.05). The level of statistical significance was determined at p < 0.05.

Table 1 shows the comparative analysis between Group I, Group II, and Group III. When Group I was compared to Group II, the mean difference was -0.167700, and when compared to Group III, the mean difference was -0.207900. Both of these differences were statistically significant, with p-values of 0.005 for CAC and 0.001 for TAC, respectively.

**Table 1:** Post hoc Tukey test results of intergroup comparison between experimental groups (apical transportation (mm)) * denotes the statistically significant value (p < 0.05). 3D, three-dimensional; CAC, conservative access cavity; TAC, traditional access cavity

Groups (I)	Groups (J)	Mean difference (I-J)	Standard error	p-value	95% CI	
					Lower bound	Upper bound
Group I: 3D-printed static guided access cavity preparation	Group II: CAC preparation	-0.167700^*^	0.048693	0.005	-0.28843	-0.04697
	Group III: TAC preparation	-0.207900^*^	0.048693	0.001	-0.32863	-0.08717
Group II: CAC preparation	Group III: TAC preparation	-0.0402	0.048693	0.691	-0.16093	0.08053

In contrast, when comparing the mean difference between CAC preparation and TAC preparation, the observed mean difference was 0.048693. This difference was not statistically significant, with a p-value of 0.691, indicating no significant difference between these two groups.

Table 2 presents the mean values for debris accumulation observed for the guided endodontic technique (GAC), CAC, and TAC preparations. The mean values were 0.928 ± 0.824 for GAC, 0.751 ± 0.495 for CAC, and 0.938 ± 0.681 for TAC. These differences were not statistically significant, with a p-value of 0.789, indicating no significant variation in debris accumulation across the different canal preparation techniques.

**Table 2:** Intergroup comparison between experimental groups (debris accumulation (mm3)) 3D, three-dimensional; CAC, conservative access cavity; NS, not significant; TAC, traditional access cavity

Debris accumulation (mm^3^)	N	Mean + SD	95% CI for mean			Sum of squares	df	Mean square	F-value	p-value
			Lower bound	Upper bound						
Group I: 3D-printed static guided access cavity preparation	10	0.928 + 0.824	0.338	1.518	Between groups	0.222	2	0.111	0.239	0.789 (NS)
Group II: CAC preparation	10	0.751 + 0.495	0.397	1.105	Within groups	12.512	27	0.463		
Group III: TAC preparation	10	0.938 + 0.681	0.45	1.425	Total	12.733	29			
Total	30	0.872 + 0.662	0.625	1.12						

Table 3 compares the GAC (Group I) with TAC (Group III) and CAC (Group II) preparations. The mean differences observed were -3.038000 and -3.902000, respectively, both of which were statistically significant, with p-values of 0.024 and 0.003. In the comparison between Group II and Group I for untouched canal surfaces, the mean difference was -0.864000. This difference was not statistically significant, with a p-value of 0.707, indicating no significant variation between these two groups.

**Table 3:** Post hoc Tukey test of intergroup comparison of untouched canal surfaces (%) between experimental groups (untouched canal surfaces (%)) * denotes the statistically significant values (p < 0.05). 3D, three-dimensional; CAC, conservative access cavity; TAC, traditional access cavity

Groups (I)	Groups (J)	Mean difference (I-J)	Standard error	p-value	95% CI	
					Lower bound	Upper bound
Group I: 3D-printed static guided access cavity preparation	Group II: CAC preparation	0.864	1.081668	0.707	-1.81791	3.54591
	Group III: TAC preparation	3.902000^*^	1.081668	0.003	1.22009	6.58391
Group II: CAC preparation	Group III: TAC preparation	3.038000^*^	1.081668	0.024	0.35609	5.71991

## Discussion

Access cavity preparation is a crucial step in nonsurgical endodontic treatment, significantly impacting the success of the procedure [15]. This step involves creating an opening in the tooth to access the root canal system. Adequate access preparation is essential for efficient instrumentation and obturation, two processes that are fundamental to successful root canal therapy. Instrumentation involves cleaning and shaping the canal to remove infected tissue and prepare it for filling, while obturation involves filling and sealing the canal to prevent bacterial recontamination and secondary infection [15].

One of the primary goals of access cavity preparation is to facilitate these processes by providing a clear and unobstructed path to the root canal. However, this must be balanced against the need to preserve as much of the tooth structure as possible. Inadvertently cutting the tooth structure while preparing an access cavity can affect the structural integrity of the tooth, making it more susceptible to fracture. Indeed, one of the major reasons for the failure of endodontic treatment is tooth fracture, which often results from the excessive loss of tooth structure [22].

In traditional endodontic preparation, achieving direct access to the canal typically requires the removal of a significant amount of tooth structure. This approach, while effective in providing clear access to the root canal, has several drawbacks. The extensive removal of tooth structure increases strain on both the crown and root surfaces, which can lead to undesirable outcomes for the tooth. These outcomes include a higher likelihood of fracture and a reduced ability to withstand masticatory forces over time [5]. Additionally, removing a large amount of tooth structure can weaken the overall tooth, making it more susceptible to future damage and reducing its longevity.

Access cavities have undergone significant improvements to minimize tooth loss and enhance fracture resistance. One such design is the CAC design, which provides access to the pulp chamber without completely removing the pulpal roof. This approach preserves the pericervical dentin, a critical factor influencing the fracture resistance of endodontically treated teeth [5,23,24]. In addition to conservative designs, another innovative method is the GAC. This technique employs a guide fabricated by integrating cone beam CT or micro-CT imaging with a surface scan of the tooth, thereby reducing the substance loss typically associated with conventional techniques [2].

In the current study, micro-CT was selected due to its reliability and minimally invasive nature for analyzing root canal preparation [25]. Preoperative and postoperative micro-CT analyses are instrumental in evaluating changes in root canal morphology, identifying untouched canal surfaces, and detecting debris accumulation without causing damage to the specimens [16].

Although previous studies have examined the fracture resistance of teeth prepared with various access cavity designs, there is a notable gap in the literature regarding apical transportation, debris accumulation, and untouched canal surfaces in different access cavity preparations [7,8]. Therefore, the present study aimed to evaluate different access cavity designs and their effects on the root canal surface, specifically focusing on apical transportation, debris accumulation, and untouched canal surfaces using micro-CT. By employing this advanced imaging technique, the study provides a detailed analysis of the impact of different access cavity designs on the internal morphology of the root canal system, offering valuable insights that could inform clinical practice and improve the outcomes of endodontic treatments.

Based on the preoperative and postoperative micro-CT images, the present study demonstrated apical transportation in all the groups tested. This transportation was found to be statistically significant in Group I (guided access cavity preparation), with negative values suggesting that the transportation was toward the distal wall of the mesial root and thus toward the furcation region. This was in contrast to Group II (CAC preparation) and Group III (TAC preparation). Notably, there was no significant difference observed between Group II and Group III. Similar results were observed in a study conducted by Silva et al., which reported a negligible level of apical transportation in the CAC group [2]. Previous studies have indicated that apical foramen transportation exceeding 0.30 mm can negatively impact the longevity of the treatment, owing to the loss of the apical seal caused by excessive dentin removal and the alteration of the original canal curvature toward the inner aspect of the tooth [20].

In the current study, no statistically significant differences in debris accumulation were observed between the experimental groups. This finding aligns with the results of a previous study by Rover et al., which reported no significant differences in the percentage of hard tissue debris accumulated following preparation between TACs and CACs [7].

The highest percentage of untouched surfaces was found in Group I (3D-guided endodontic access cavity preparations) and the least in Group III (TAC preparation), with a statistically significant difference observed between the two. Between Group II (CAC preparation) and Group III, Group II had more untouched surfaces than Group III, also with a statistically significant difference. These results correlate with a study conducted by Lima et al., which found that TAC preparation showed the least percentage of untouched canal surfaces compared to ultraconservative cavity preparation [25].

The higher percentage of untouched canal surfaces in Group I and Group II could be attributed to the absence of straight-line access to the canals and the decreased efficiency of the instruments used.

In the present study, TruNatomy files were used for preparing the canals due to their regressive taper, slim design, and off-centered parallelogram cross-section [23,25]. These features allow for better preservation of tooth structure during the preparation of the root canal. Silva et al. reported that TruNatomy files were equally efficient as Protaper Gold files in assessing apical transportation and untouched surfaces in minimally invasive cavity designs [26].

The present study has several limitations related to methodology and material selection. First, it focused solely on the mesiobuccal canals of mandibular first molars, chosen for their specific cross-sectional shape and their impact on instrumentation efficiency. Previous studies, however, have evaluated distal canals with oval-shaped cross-sections, which may affect result comparability. Second, the study did not assess the ability of the irrigant to penetrate the canal, a crucial factor for effective root canal treatment as it aids in removing bacterial contaminants and debris. This omission may influence the final outcomes and interpretation of the study results.

It can be said that, compared to other access cavity preparations examined, the 3D-printed static-guided access cavity preparation did not provide a discernible advantage. TAC preparations performed well relative to the other groups studied. Despite conserving significant tooth structure, CAC preparation did not show significant differences from TAC preparation in the assessed parameters.

## Conclusions

Within the limitations of this study, it can be concluded that 3D-printed static-guided access cavity designs should be used only when absolutely necessary. TAC preparation demonstrated better results in the tested parameters; however, the difference compared to conservative access preparation was not statistically significant. Although the conservative approach aims to preserve root dentin, it may not always offer clear advantages in treatment outcomes. It is important to balance tooth structure conservation with adequate access for effective root canal instrumentation.

In light of these findings, practitioners should consider the specific clinical scenario when selecting an access cavity design. TAC preparation showed reliable results and practical advantages in clinical practice. The choice should balance preserving tooth structure with ensuring adequate access for successful endodontic treatment. While CAC designs aim to conserve tooth structure, TAC preparation remains a robust and effective option for achieving optimal outcomes.
